# Properties of Nav1.8^ChR2^-positive and Nav1.8^ChR2^-negative afferent mechanoreceptors in the hindpaw glabrous skin of mice

**DOI:** 10.1186/s13041-023-01015-z

**Published:** 2023-03-07

**Authors:** Akihiro Yamada, Ayaka I. Yamada, Jennifer Ling, Hidemasa Furue, Wenqin Luo, Jianguo G. Gu

**Affiliations:** 1 Department of Anesthesiology and Perioperative Medicine, School of Medicine, University of Alabama at Birmingham, Birmingham, AL 35294 USA; 2 Department of Neurophysiology, Hyogo Medical University, Nishinomiya, 663-8501 Japan; 3 Department of Neuroscience, Perelman School of Medicine, University of Pennsylvania, Philadelphia, PA 19104 USA

**Keywords:** Nav1.8, Optogenetics, Opto-tagged single-fiber recording, High threshold mechanoreceptors (HTMRs), Low threshold mechanoreceptors (LTMRs), Touch, Mechanical pain, Hindpaw glabrous skin

## Abstract

Nav1.8-positive afferent fibers are mostly nociceptors playing a role in mediating thermal and mechanical pain, but mechanoreceptors within these afferents have not been fully investigated. In this study, we generated mice expressing channel rhodopsin 2 (ChR2) in Nav1.8-positive afferents (Nav1.8^ChR2^), which showed avoidance responses to mechanical stimulation and nocifensive responses to blue light stimulation applied to hindpaws. Using ex vivo hindpaw skin-tibial nerve preparations made from these mice, we characterized properties of mechanoreceptors on Nav1.8^ChR2^-positive and Nav1.8^ChR2^-negative afferent fibers that innervate the hindpaw glabrous skin. Of all Aβ-fiber mechanoreceptors, small portion was Nav1.8^ChR2^-positive. Of all Aδ-fiber mechanoreceptors, more than half was Nav1.8^ChR2^-positive. Of all C-fiber mechanoreceptors, almost all were Nav1.8^ChR2^-positive. Most Nav1.8^ChR2^-positive Aβ-, Aδ-, and C-fiber mechanoreceptors displayed slowly adapting (SA) impulses in response to sustained mechanical stimulation, and their mechanical thresholds were high in the range of high threshold mechanoreceptors (HTMRs). In contrast, sustained mechanical stimulation applied to Nav1.8^ChR2^-negative Aβ- and Aδ-fiber mechanoreceptors evoked both SA and rapidly adapting (RA) impulses, and their mechanical thresholds were in the range of low threshold mechanoreceptors (LTMRs). Our results provide direct evidence that in the mouse glabrous skin, most Nav1.8^ChR2^-negative Aβ-, Aδ-fiber mechanoreceptors are LTMRs involving in the sense of touch, whereas Nav1.8^ChR2^-positive Aβ-, Aδ-, and C-fiber mechanoreceptors are mainly HTMRs involving in mechanical pain.

## Introduction

Mechanical stimuli, such as a gentle touch or a strong pinch to the skin, activate mechanoreceptors to signal the sense of touch or mechanical pain, respectively. Mechanoreceptors are classified into several subtypes based on their distinctive mechanical thresholds (LTMRs, HTMRs), afferent fiber conduction velocities (Aβ-, Aδ-, and C-fibers), and impulse adaptation types (slowly adapting, SA; rapidly adapting, RA) to sustained mechanical stimulation [11]. A gentle touch is transduced by low threshold mechanoreceptors (LTMRs) consisting terminals of non-nociceptive afferents, whereas a strong pinch activates high threshold mechanoreceptors (HTMRs) on nociceptive afferent endings [1, 11, 27]. In mouse glabrous skin, there are at least two main types of LTMRs, Aβ-fiber slowly adapting type 1 LTMRs (Aβ-fiber SA1-LTMRs) that are consist of Merkel cell-neurite complex, and Aβ-fiber rapidly adapting type 1 LTMRs (Aβ-fiber RA1-LTMRs) that terminate in Meissner’s corpuscles [1, 11, 27]. In contrast to LTMRs, HTMRs are present as free nerve endings of nociceptive afferent fibers in the glabrous skin, and they usually display SA impulses in response to sustained mechanical stimulation [11]. HTMRs are believed to be mainly nerve endings of nociceptive C- and Aδ-fibers [4, 6, 7], but Aβ-fiber HTMRs are also present in the skin and may serve as nociceptors for mechanical tissue damage [4, 9]. Properties of HTMRs, particularly Aβ-fiber HTMRs, have not been well characterized because they don’t have well defined structures and molecular markers.

Transgenic mouse lines expressing Cre recombinase under the control of the promoters of specific sensory molecules allow the expression of fluorescent proteins (e.g. GFP) in distinct subpopulation of afferents. This genetic approach can label afferent subpopulations for structural and functional studies [13]. The genetic reporter mice generated with Cre technique in combination with electrophysiology has allowed to characterize properties of different types of mechanoreceptors. Cre mouse lines generated for genetic labeling of LTMRs include TrkC^creER^ for Aβ-fiber SA1-LTMR of Merkel cell-neurite complex in the skins [5], Ret^CreER^ and TrkB^CreER^ for Aβ-fiber RA1-LTMR of Meissner corpuscles in glabrous skin [15, 18]; and TH^CreER^ for C-fiber LTMRs in hair follicles [14]. For nociceptors including HTMRs, several Cre mouse lines have been generated to allow to genetically label subpopulations of nociceptors for studies on properties and functions of these nociceptors. Cre mouse lines for labeling nociceptors include Mrgprd^cre^, CGRP^cre^, Nav1.8^cre^, and others [11, 19]. Nav1.8 are voltage-gated Na^+^ channels largely expressed in small-sized C-fiber nociceptors that are involved in both mechanical and thermal nociception [2, 3]. Nav1.8^cre^ has also been found in a large population of C-fiber nociceptors but they are also observed in some LTMRs [20]. A recent study has used CGRP^cre^, TRPV1^cre^, and Nav1.8^cre^ mouse lines to characterize electrophysiological properties and immunochemical profiles of CGRP^cre^-positive, TRPV1^cre^-positive, and Nav1.8^cre^-positive dorsal root ganglion (DRG) neurons [19]. The study shows that Nav1.8^cre^ mouse line labels almost all C-fibers, whereas CGRP^cre^ and TRPV1^cre^ mouse lines label subpopulations of nociceptive C-afferents. Interestingly, all three Cre mouse lines also genetically label afferent fibers that appear to give rise to Aδ-fiber HTMRs and Aβ-fiber HTMRs [19]. However, the properties of putative Aδ-fiber HTMRs and Aβ-fiber HTMRs in these transgenic mice have not been characterized electrophysiologically.

These transgenic Cre mice can also be used to express channel rhodopsin 2 (ChR2) in distinct subpopulations of afferents. This allows to perform optogenetic and opto-tagged electrophysiological studies on properties of different mechanoreceptors. For example, a recent study have used NPY2r^Cre+/ChR2+^ mice to optogenetically label NPY2r-positive A-fiber HTMRs for investigating their electrophysiological properties and functions in acute mechanical pain [4]. In the present study, we have used Nav1.8^cre^ mice [24] to drive the expression of ChR2 in Nav1.8-positive afferents (Nav1.8^ChR2^-positive fibers), and combined optogenetic and electrophysiological approaches to study the properties of Nav1.8^ChR2^-positive and Nav1.8^ChR2^-negative afferent mechanoreceptors in the hindpaw glabrous skin of Nav1.8^ChR2^ mice.

## Materials and methods

### Animals

Nav1.8^ChR2^ mice were generated by crossing Nav1.8^cre^ mice with Ai32 (RCL-ChR2(H134R)/EYFP) mice. Nav1.8^cre^ mice were gifts from Dr. John Wood at University College London and transferred to us from Dr. Stephen Waxman’s lab at Yale University. Ai32 mice were purchased from Jackson Labs. Animal care and use conformed to NIH guidelines for care and use of experimental animals. Experimental protocols were approved by the Institutional Animal Care and Use Committee (IACUC) at the University of Alabama at Birmingham.

### Behavioral assessment

*Cotton swab test* The cotton swab test was performed in a manner described previously [10]. In brief, each testing mouse was covered by a glass cup (7 cm in diameter and 8.5 cm in height) on an elevated platform with a perforated metal floor (Ugo Basile). Animals were acclimatized to the environment for approximately 1 h. Each cotton swab was made by a piece of cotton that was glued onto a wood stick with the cotton part approximately 12 mm in length. The hindpaw of mice was brushed by the cotton swab in the heel-to-toe direction for 5 times, and the frequency of avoidance responses were measured.*von Frey test* Each testing mouse was covered by a glass cup (7 cm in diameter and 8.5 in height) on an elevated platform with a perforated metal floor (Ugo Basile). Animals were habituated to the environment for approximately 1 h. The plantar side of the hindpaw was poked by calibrated von Frey filaments (North coast medical, NC12775-99). The von Frey filaments used are 0.02, 0.04, 0.07, 0.16, 0.4, 0.6, 1.0, 1.4 g, and the 50% paw withdrawal thresholds were determined using the Up-Down method [8]. In a different von Frey test, the plantar side of hindpaw was poked with 0.07-g and 0.4-g von Frey filaments each for 10 times with intervals between stimuli being 1 to 2 min, and the percent of avoidence responses were determined.

*Light stimulation* Blue laser beam was applied to the planter surface with an optical fiber (diameter: 0.2 mm: Laserglow technologies). The light intensities were calibrated with an optical power and energy meter (PM100D, Thorlab). The light duration was 50 ms and intensities were 1, 2.5, 5, 10, 20, 50, 100 mW/mm^2^. The light evoked responses were scored as 0, no response; 1, hindpaw lift; 2, hindpaw flinch, flutter, and/or hold; 3, jump, vocalization, lick, and/or guard.

### Ex vivo skin-nerve preparations

Nav1.8^ChR2^ mice of both males and females aged 8–11 weeks were used. Animals were anesthetized with 5% isoflurane and then sacrificed by decapitation. The hindpaw glabrous skin including plantar and finger regions together with medial planter nerve and tibial nerve before the branch from sciatic nerves were dissected out. The skin-nerve preparation was then placed in a Sylgard Silicone-coated bottom of a 60-mm recording chamber. The fat, muscle and connective tissues on the nerves and the skin were carefully removed with a pair of forceps. The skin was affixed to the bottom of the chamber by tissue pins with epidermis side facing up, and the nerve bundle was affixed by a tissue anchor in the same recording chamber. The cutting end of the nerve bundle was briefly exposed to a mixture of 0.05% dispase II plus 0.05% collagenase for 30–60 s, and the enzymes were then washed off by the normal Krebs solution (see below). This gentle enzyme treatment was to help separating individual afferent fibers at the cutting end of the nerve bundle so that a single fiber could be aspirated into the recording electrode and pressure-clamped for single-fiber recordings (see below). The recording chamber was then mounted on the stage of the Olympus BX51WI upright microscope. The skin-nerve preparation was superfused with a normal Krebs bath solution that contained (in mM): 117 NaCl, 3.5 KCl, 2.5 CaCl_2_, 1.2 MgCl_2_, 1.2 NaH_2_PO_4_, 25 NaHCO_3_, and 11 glucose (pH 7.3 and osmolarity 325 mOsm) and was saturated with 95% O_2_ and 5% CO_2_. The Krebs bath solution in the recording chamber was maintained at 28–32 °C during experiments.

### Pressure-clamped single-fiber recordings

The pressure-clamped single-fiber recording was performed in the similar manner described in our previous studies [22, 23] to measure impulses evoked by blue light and mechanical stimulation. In brief, recording electrodes for pressure-clamped single-fiber recordings were made by thin-walled borosilicate glass tubing without filament (inner diameter 1.12 mm, outer diameter 1.5 mm, World Precision Instruments, Sarasota, FL). They were fabricated by using P-97 Flaming/Brown Micropipette Puller (Sutter Instrument Co., Novato, CA) and the tip of each electrode was fire polished by a microforge (MF-900, Narishige) to final size of 4 to 8 μm in diameter. The recording electrode was filled with Krebs bath solution, mounted onto an electrode holder which was connected to a high-speed pressure-clamp (HSPC) device (ALA Scientific Instruments, Farmingdale, NY) for fine controls of intra-electrode pressures. Under a 40 × objective, the end of individual afferent nerve was visualized and separated by applying a low positive pressure (~ 10 mmHg or 0.19 Psi) from the recording electrode. The end of a single nerve fiber was then aspirated into the recording electrode by a negative pressure at approximately 10 mmHg. Once the end of the nerve fiber entered into the recording electrode in approximately 10 µm, the electrode pressure was readjusted to − 3 ± 2 mmHg and maintained at the same pressure throughout the experiment. Nerve impulses on the single afferent fiber were recorded under the I_0_ configuration and amplified using a Multiclamp 700B amplifier (Molecular Devices, Sunnyvale, CA). Electrical signals were amplified 500 times and sampled at 25 kHz with AC filter at 0.1 Hz and Bessel filter at 3 kHz under AC membrane mode (Digidata 1550B, Molecular Devices). All experiments were performed at 30 ± 2 °C.

To determine conduction velocity of recorded afferent fibers, action potential (AP) impulses were initiated by electrical stimulation using a bipolar stimulation electrode positioned on the tibial nerve bundle. The distance between the electrical stimulation site and the recording site was approximately 12 mm. Electrical stimuli were monophasic square pulses that were generated by an electronic stimulator (Master-9, A.M.P.I, Israel) with a stimulation isolator (ISO-Flex, A.M.P.I, Israel) and delivered to the stimulation electrode. The duration of each stimulation pulse was 200 μs for A-fibers and 2 ms for C-fibers, and the stimulation intensities for evoking impulses were 0.3–1.7 mA for A-fiber and 0.65–2.5 mA for C-fibers.

### Mechanical and light stimulation

For a recorded afferent fiber, its mechanosensitive receptive field in the hindpaw glabrous skin was first searched using a glass rod. Poking with the glass rod at the mechanosensitive receptive field of the recorded afferent fiber would result in the detection of APs by the recording electrode. In the present study, all data were collected from mechanosensitive receptive sites, i.e., mechanoreceptors in the hindpaw glabrous skin. Once a mechanoreceptor was identified, mechanical stimulation was applied to the same receptive field with a force-calibrated mechanical indenter (300C-I, Aurora Scientific Inc., Ontario, Canada) to determine mechanical thresholds. The tip size of the indenter was 0.8 mm in diameter. The indenter was connected to a Digidata 1550B Digitizer to allow generating ramp-and-hold mechanical stimulation using the pClamp 11 software. Prior to the application of mechanical stimulation, the tip of the indenter was lowered to the surface of the receptive field with a 10-mN force and then the 10-mN force was canceled to 0 so that the tip of the indenter was just in contact with the receptive field surface. Under the force control module, ramp-and-hold mechanical stimuli were applied to the mechanoreceptor of the glabrous skin. The step force commanders were calibrated by applying indenter at finger tips, paw pads and other areas of plantar skin, and the actual forces after the calibration were used in experiments. The ramp-and-hold force steps were at 0, 5, 30, and 80 mN. The duration of the ramp (dynamic phase) was 10 ms, and the duration of the holding (static phase) was 0.98 s. The minimal force at which AP impulses was elicited was defined as intender mechanical threshold of the mechanoreceptors. In a different set of experiments mechanical stimulation was applied using von Frey filaments to vertically poke the glabrous skin. The von Frey mechanical thresholds were determined by mechanical stimulation with von Frey filaments (0.08 ~ 6 g) onto mechanoreceptors.

To determine whether a mechanoreceptor was from Nav1.8^ChR2^-positive or Nav1.8^ChR2^-negative afferent fibers, the same mechanosensitive receptive field was stimulated by a blue LED light (Thorlab; M455L4, 455 nm) to test light sensitivity. A mechanoreceptor was from Nav1.8^ChR2^-positive afferent fibers if light stimulation evoked impulses. Otherwise, the mechanoreceptor was from light-insensitive or Nav1.8^ChR2^-negative afferent fibers. The blue Light was applied through a 40 × objective to a mechanoreceptor with a 1-s light stimulation pulse at the intensity of 50 mW. Afferent impulses evoked by mechanical and light stimulation were recorded using the pressure-clamped single-fiber recordings, and signals were amplified by the Multiclamp 700B amplifier and sampled at 25 kHz with band path filter between 0.1 Hz and 3 kHz on AC recording mode.

### Data analysis

Electrophysiological data were analyzed using Clampfit 11 (Molecular Devices, Sunnyvale, CA, USA). Data were collected from 27 male and 13 female animals and were aggregated together for data analysis since no statistically significant difference was found between male and female animals. To confirm that impulses evoked by blue light and mechanical stimulators (indenter or von Frey) are generated from the same receptive field, the amplitudes and shapes of the impulses evoked by both blue light and mechanical stimulators were compared to ensure that mechanically evoked impulses matched the light-evoked impulses. Conduction velocity (CV) was calculated by the distance between stimulation site and recording site divided by the time latency for eliciting an AP impulse following electrical stimulation. Afferent fibers were classified as Aβ-fibers with CV > 9 m/s, Aδ-fiber with CV between 1.2 and 9 m/s, and C-fiber with CV < 1.2 ms [9, 26]. All data analyses were performed using Graph Pad Prism (version 8). Unless otherwise indicated, all data were reported as individual observations and/or mean ± SEM of *n* independent observations. Statistical significance was evaluated using the Kruskal–Wallis (nonparametric) test with Dunn’s post hoc tests for multiple group comparison, Mann–Whitney (nonparametric) test or Student’s t tests for two group comparison. Differences were considered to be significant with *p < 0.05, **p < 0.01, ***p < 0.001, and not significant (ns) with p ≥ 0.05.

## Results

### Behavioral responses to mechanical and light stimulation applied to the hindpaw plantar region of Nav1.8^ChR2^ mice

**Fig. 1:**
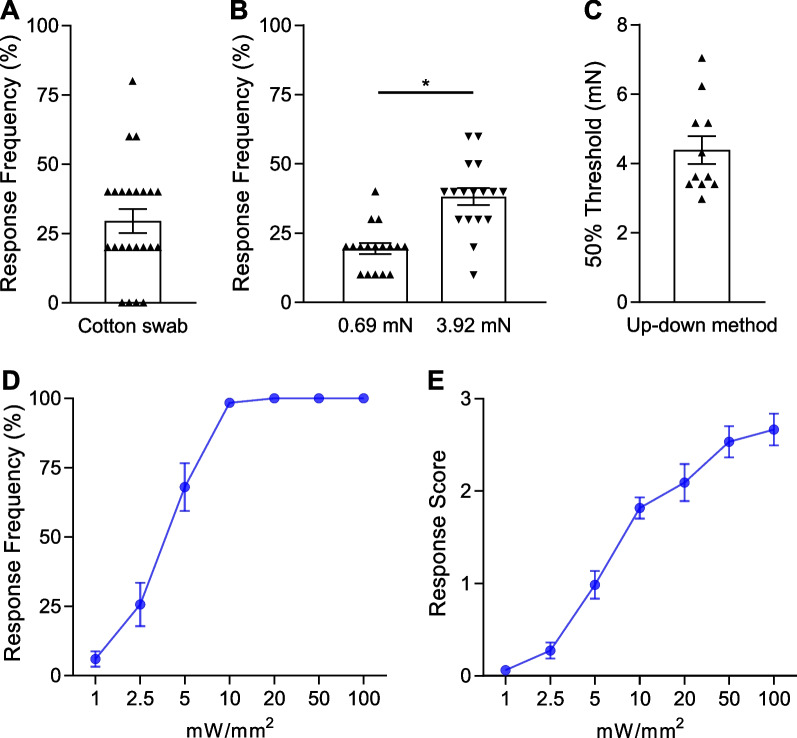
Behavioral responses to mechanical and light stimulation at the hindpaws of Nav1.8^ChR2^ mice. **A** Frequency of hindpaw avoidance in response to cotton swab strikes in Nav1.8^ChR2^ mice (n = 23). **B** Frequency of hindpaw avoidance in response to mechanical stimulation by 0.69-mN (left bar, n = 17) and 3.92-mN von Frey filaments (right bar, n = 17) in Nav1.8^ChR2^ mice. **C** The 50% threshold of hindpaw avoidance responses assessed by von Frey filaments using the up-down method in Nav1.8^ChR2^ mice (n = 11). **D** Frequency of hindpaw avoidance in response to the stimulation by blue laser light at intensities from 1 to 100 mW/mm^2^ (n = 5 to 22). **E** Scores of nocifensive responses induced by blue laser light at intensities from 1 to 100 mW/mm^2^ (n = 5 to 22). Data represent individual observations and/or mean ± SEM, *p < 0.05

We crossed Nav1.8^Cre^ mice with Ai32 (RCL-ChR2(H134R)/EYFP) mice to generate Nav1.8^cre+^;ChR2-EYFP^loxP/+^ mouse line, hereafter termed Nav1.8^ChR2^. We first examined behavioral responses of Nav1.8^ChR2^ mice to mechanical stimulation by cotton swabs and von Frey filaments applied to the hindpaw plantar regions of Nav1.8^ChR2^ mice. For the stimulation with cotton swabs, animals withdrew their hindpaws at the frequency of 30 ± 4% (n = 23, Fig. 1A) in response to the gentle strikes of their hindpaw plantar regions with cotton swabs. For the hindpaw stimulation with von Frey filaments of 0.69-mN force and 3.92-mN force, animals withdrew their hindpaws at the frequency of 19 ± 2% and 38 ± 3% (n = 17, Fig. 1B), respectively. We also used the up-down method with von Frey filaments to determine 50% response thresholds. The 50% response thresholds were 4.39 ± 0.4 mN (n = 11, Fig. 1C) for evoking hindpaw withdraw responses.

We examined behavioral responses to blue light stimulation applied to the hindpaw plantar region of Nav1.8^ChR2^ mice. In this set of experiments, a blue laser beam was applied to the hindpaw plantar region with the light intensity ranged from 1 to 100 mW/mm^2^. Light stimulation evoked nocifensive responses including paw lift, flinch, flutter, hold, jump, vocalization, lick, and guard. We first analyzed response frequency without considering the types of responses. The response frequencies were increased in a light stimulation intensity-dependent manner from a narrow range of 1 to 10 mW/mm^2^ (n = 22), and the response frequency quickly reached 100% with light intensity at 10 mW/mm^2^ (n = 22), and remained the ceiling effect at 20 mW/mm^2^ (n = 11), 50 mW/mm^2^ (n = 5), and 100 mW/mm^2^ (n = 6) (Fig. 1D). We next analyzed nocifensive responses by scoring light-evoked responses, and determined relationship between light stimulation intensity and response scores. The following criteria were used for the response scores: score 0, no response; score 1, hindpaw lift; score 2, hindpaw flinch, flutter, and/or hold; score 3, jump, vocalization, lick and/or guard hindpaw. As shown in Fig. 1E, the nocifensive scores were increased in a light intensity-dependent manner from 1 to 100 mW/mm^2^ (1 to 10 mW/mm^2^, n = 22; 20 mW/mm^2^, n = 22; 50 mW/mm^2^, n = 5; 100 mW/mm^2^, n = 6). Thus, the hindpaw plantar regions of Nav1.8^ChR2^ mice appeared to have normal mechanical sensitivities and showed graded nocifensive behavioral responses to the increased light stimulation intensity.

### Characterization of Nav1.8^ChR2^-positive mechanoreceptors

**Fig. 2:**
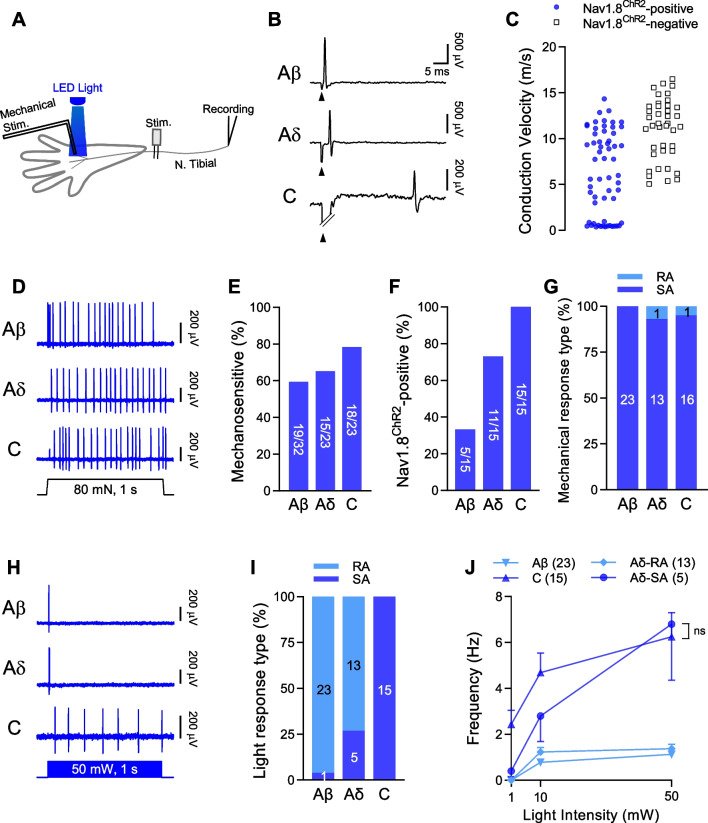
Properties of Nav1.8^ChR2^-positive mechanoreceptors in the hindpaw glabrous skin of Nav1.8^ChR2^ mice. **A** Schematic diagram illustrate the experimental setting for characterizing Nav1.8^ChR2^-positive, i.e., light-sensitive, mechanoreceptors in the hindpaw glabrous skin of the ex vivo skin-nerve preparation made from Nav1.8^ChR2^ mice. AP impulses evoked by mechanical stimulation and light stimulation at the same receptive fields were recorded using the pressure-clamped single-fiber recording technique. **B** Sample traces show examples of Aβ-, Aδ- and C-fiber mechanoreceptors whose conduction velocities (CV) were determined by electrical stimulation. Arrowhead in each panel indicates electrical stimulation artifact, and the latency between the stimulation artifact and AP impulse was used to calculating CV. **C** Plots of CV of individual Nav1.8^ChR2^-positive mechanoreceptors (solid circles) and Nav1.8^ChR2^-negative mechanoreceptors (open squares). **D** Sample traces show slowly adapting (SA) impulses evoked by an 80-mN ramp-and-hold stimulation in a Nav1.8^ChR2^-positive Aβ-fiber mechanoreceptor (top), a Nav1.8^ChR2^-positive Aδ-fiber mechanoreceptor (middle), and a Nav1.8^ChR2^-positive C-fiber mechanoreceptors. **E** Percent of Nav1.8^ChR2^-positive afferent fibers that are mechanosensitive. Numbers in the bar represent Nav1.8^ChR2^-positive mechanoreceptors over total Nav1.8^ChR2^-positive afferent fibers tested. **F** Percent of mechanoreceptors that are Nav1.8^ChR2^-positive afferent fibers. Numbers in the bar represent Nav1.8^ChR2^-positive mechanoreceptors over total mechanoreceptors. **G** Percent of Nav1.8^ChR2^-positive mechanoreceptors displaying SA and rapidly adapting (RA) impulses. Numbers in the bar represent numbers of recordings. **H** Examples of AP impulses evoked by blue LED light (50 mW, 1 s) applied to the mechanosensitive receptive field of an Aβ- (top), an Aδ- (middle), and a C-fiber mechanoreceptor (bottom). **I** Percent of recordings showing SA or RA for the Nav1.8^ChR2^-positive Aβ-, Aδ-, and a C-mechanoreceptor. Numbers in the bar represent numbers of recordings. **J** Frequencies of AP impulses evoked by blue light at 1, 10 and 50 mW in SA Aδ-fiber SA-mechanoreceptors (n = 5), C-fiber SA-mechanoreceptors (n = 15), Aβ-fiber RA-mechanoreceptors (n = 23), and Aδ-fiber RA-mechanoreceptors (n = 13). Data represent individual observations or mean ± SEM

We characterized properties of Nav1.8^ChR2^-positive mechanoreceptors by using the ex vivo plantar skin-tibial nerve preparation. In this set of experiments, Nav1.8^ChR2^-positive mechanoreceptors in the plantar skin were identified by both mechanical stimulation and blue light stimulation. A Nav1.8^ChR2^-positive mechanoreceptor was a receptive field where both mechanical stimulation and blue light stimulation evoked AP impulses which individually had identical shape. In this set of experiments, mechanical stimulation-evoked AP impulses and light stimulation-evoked impulses at the same receptive fields were recorded using the pressure-clamped single-fiber recording technique (Fig. 2A). Furthermore, the conduction velocity (CV) of each recorded afferent fiber was measured, and the afferent fibers were classified into Aβ-fibers with CV > 9 m/s [9, 26], Aδ-fibers with CV between 9 and 1.2 m/s, and C-fibers with CV < 1.2 m/s (Fig. 2B, C). In addition to Nav1.8^ChR2^-positive mechanoreceptors, we collected data from Nav1.8^ChR2^-negative (light-insensitive) mechanoreceptors. Figure 2C shows the CV of individual Nav1.8^ChR2^-positive mechanoreceptors and Nav1.8^ChR2^-negative mechanoreceptors. Nav1.8^ChR2^-poistive mechanoreceptors included Aβ-, Aδ-, and C-fibers. Our recordings with Nav1.8^ChR2^-negative mechanoreceptors identified Aβ-, Aδ-, but not C-fibers (Fig. 2C). Of all Nav1.8^ChR2^-positive Aβ-fibers tested, 59% of them were mechanosensitive (Fig. 2D, E). Of all Nav1.8^ChR2^-positive Aδ-fiber, 65% of them were mechanosensitive (Fig. 2D, E). Of all Nav1.8^ChR2^-positive C-fibers, 78% of them were mechanosensitive (Fig. 2D, E). When Nav1.8^ChR2^-positivity was examined for the mechanoreceptors, of all Aβ-fiber mechanoreceptors, 33% of them were Nav1.8^ChR2^-positivity (Fig. 2F). Of all Aδ-fiber mechanoreceptors, 73% of them were Nav1.8^ChR2^-positivity, and of all C-fiber mechanoreceptors (Fig. 2F), 100% of them were Nav1.8^ChR2^-positivity (Fig. 2F). For Nav1.8^ChR2^-positive Aβ-, Aδ-, and C-fiber mechanoreceptors, mechanical stimulation by ramp-and-hold indentation applied to the receptive fields in the hindpaw regions evoked slowly adapting (SA) type of impulses in almost all recordings (Fig. 2D, G).

We examined AP impulses evoked by sustained blue light stimulation (50 mW, 1 s) applied to the mechanosensitive receptive fields. The sustained light stimulation evoked rapidly adapting (RA) impulses in almost all Nav1.8^ChR2^-positive Aβ-fiber mechanoreceptors (Fig. 2H, I, n = 23/24). The light stimulation evoked RA impulses in majority (n = 13/18) and SA impulses in minority (Fig. 2H, I, n = 5/18) of Nav1.8^ChR2^-positivive Aδ-fiber mechanoreceptors. In contrast, the light stimulation evoked SA impulses in all Nav1.8^ChR2^-positive C-fiber mechanoreceptors (Fig. 2H&I, n = 15/15). The light-evoked SA impulses showed increased frequency in a light stimulation intensity-dependent manner for both Nav1.8^ChR2^-positive Aδ-fiber SA-mechanoreceptors (Fig. 2J, n = 5) and Nav1.8^ChR2^-positive C-fiber mechanoreceptors (Fig. 2J, n = 15). On the other hand, the light-evoked impulses in Nav1.8^ChR2^-positive Aβ-fiber RA-mechanoreceptors (n = 23) and Nav1.8^ChR2^-positive Aδ-fiber RA-mechanoreceptors (n = 13) did not shown large enhancement of impulse frequency with increased stimulation intensity.

### Properties of Nav1.8^ChR2^-positive and Nav1.8^ChR2^-negative Aβ-fiber mechanoreceptors

**Fig. 3:**
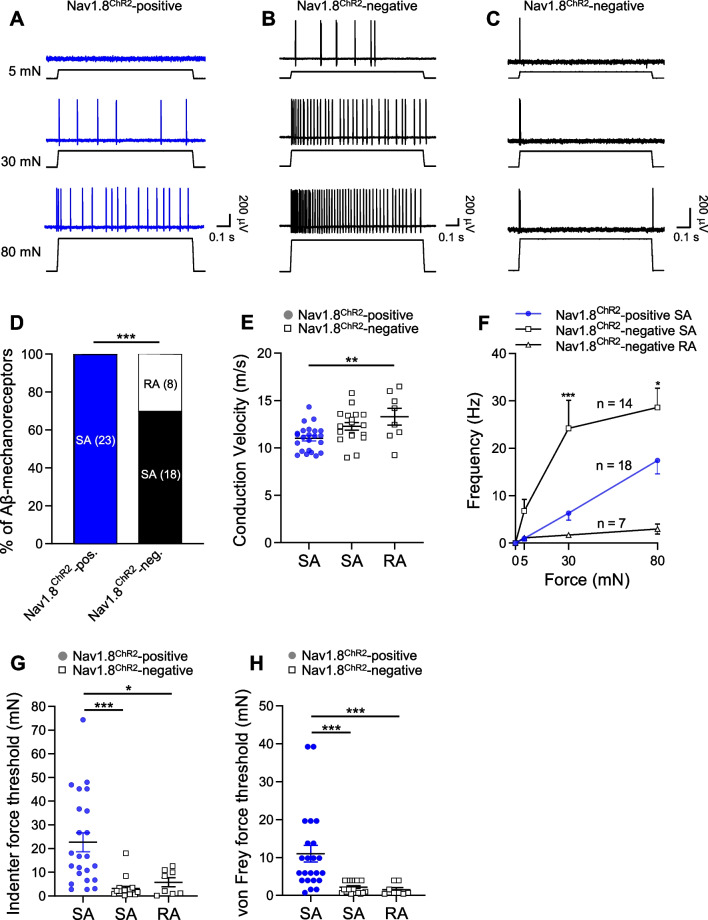
Comparison of properties between Nav1.8^ChR2^-positive and Nav1.8^ChR2^-negative Aβ-mechanoreceptors. **A** Sample traces show SA AP impulses evoked by indentations (5, 30, and 80 mN) in a Nav1.8^ChR2^-positive Aβ-fiber SA-mechanoreceptor. B&C) Two sets of sample traces show SA (**B**) and RA (**C**) AP impulses evoked by indentations (5, 30, and 80 nN) in a Nav1.8^ChR2^-negative Aβ-fiber SA-mechanoreceptors (**B**) and a Nav1.8^ChR2^-negative Aβ-fiber RA-mechanoreceptors. **D** Percent of Nav1.8^ChR2^-positive and Nav1.8^ChR2^-negative Aβ-mechanoreceptors that display SA or RA impulses. Recording numbers are indicated in the bars. **E** Conduction velocity of Nav1.8^ChR2^-positive Aβ-fiber SA-mechanoreceptors, Nav1.8^ChR2^-negative Aβ-fiber SA-mechanoreceptors, and Nav1.8^ChR2^-negative Aβ-fiber RA-mechanoreceptors. **F** Frequency of impulses evoked by different indentation forces in Nav1.8^ChR2^-positive Aβ-fiber SA-mechanoreceptors, Nav1.8^ChR2^-negative Aβ-fiber SA-mechanoreceptors, and Nav1.8^ChR2^-negative Aβ-fiber RA-mechanoreceptors. **G**, **H** Indenter (**G**) and von Frey (**H**) mechanical force thresholds for evoking AP impulses in Nav1.8^ChR2^-positive Aβ-fiber SA-mechanoreceptors (n = 23), Nav1.8^ChR2^-negative Aβ-fiber SA-mechanoreceptors (n = 18), and Nav1.8^ChR2^-negative Aβ-fiber RA-mechanoreceptors (n = 8). Data represent individual observations and/or mean ± SEM, *p < 0.05, **p < 0.01, ***p < 0.001

We compared properties of Nav1.8^ChR2^-positive Aβ-fiber mechanoreceptors with those of Nav1.8^ChR2^-negative Aβ-fiber mechanoreceptors. Ramp-and-hold mechanical stimulation evoked SA impulses in all Nav1.8^ChR2^-positive Aβ-fiber mechanoreceptors (Fig. 3A, D), but evoked both SA (18/26, Fig. 3B, D) and RA (n = 8/26, Fig. 3C, D) impulses in Nav1.8^ChR2^-negative Aβ-fiber mechanoreceptors. The differences in adapting types were significant between Nav1.8^ChR2^-positive Aβ-fiber mechanoreceptors and Nav1.8^ChR2^-negative Aβ-fiber mechanoreceptors (p < 0.001, Fig. 3D). Conduction velocities of Nav1.8^ChR2^-positive Aβ-fiber SA-mechanoreceptors, Nav1.8^ChR2^-negative Aβ-fiber SA-mechanoreceptors, and Nav1.8^ChR2^-negative Aβ-fiber RA-mechanoreceptors were compared (Fig. 3E). Conduction velocities of Nav1.8^ChR2^-positive Aβ-fiber SA-mechanoreceptors was 11.0 ± 0.3 m/s (n = 14), significantly slower than the Nav1.8^ChR2^-negative Aβ-fiber RA-mechanoreceptors (13.3 ± 0.9 m/s, n = 8, p < 0.01, Fig. 3E). AP impulse frequency largely increased with enhanced mechanical indentation force in both Nav1.8^ChR2^-positive Aβ-fiber SA-mechanoreceptors (n = 14, Fig. 3F) and Nav1.8^ChR2^-negative Aβ-fiber SA-mechanoreceptors (n = 18, Fig. 3F). However, Nav1.8^ChR2^-positive Aβ-fiber SA-mechanoreceptors displayed relatively lower AP impulse frequency (n = 14) in comparison with Nav1.8^ChR2^-negative Aβ-fiber SA-mechanoreceptors (n = 18) (Fig. 3F). For Nav1.8^ChR2^-negative Aβ-fiber RA-mechanoreceptors, there was minimal change in AP impulse frequency with increased indentation forces (n = 7) (Fig. 3F).

We examined mechanical thresholds for evoking AP impulses in Nav1.8^ChR2^-positive Aβ-fiber mechanoreceptors and Nav1.8^ChR2^-negative Aβ-fiber mechanoreceptors. Nav1.8^ChR2^-positive Aβ-fiber SA-mechanoreceptors had significantly higher mechanical thresholds in comparison with Nav1.8^ChR2^-negative Aβ-fiber SA-mechanoreceptors and Nav1.8^ChR2^-negative Aβ-fiber RA-mechanoreceptors as tested by both mechanical indenter (Fig. 3G) and von Frey filaments (Fig. 3H). For the test with mechanical indenter, the force threshold of Nav1.8^ChR2^-positive Aβ-fiber SA-mechanoreceptors was 22.7 ± 4.0 mN (n = 23), significantly higher than the force threshold of Nav1.8^ChR2^-negative Aβ-fiber SA-mechanoreceptors (3.2 ± 1.0 mN, n = 18, p < 0.001) and Nav1.8^ChR2^-negative Aβ-fiber RA-mechanoreceptors (5.8 ± 1.8 mN, n = 8, p < 0.05) (Fig. 3G). For the test with von Frey filaments, the force threshold of Nav1.8^ChR2^-positive Aβ-fiber SA-mechanoreceptors was 11.0 ± 2.2 mN (n = 23), approximately five times higher than those of Nav1.8^ChR2^-negative Aβ-fiber SA mechanoreceptors (2.1 ± 0.4 mN, n = 18, p < 0.001) and Nav1.8^ChR2^-negative Aβ-fiber RA-mechanoreceptors (2.2 ± 0.7 mN, n = 8, p < 0.001) (Fig. 3H).

### Properties of Nav1.8^ChR2^-positive and Nav1.8^ChR2^-negative Aδ-fiber mechanoreceptors

**Fig. 4:**
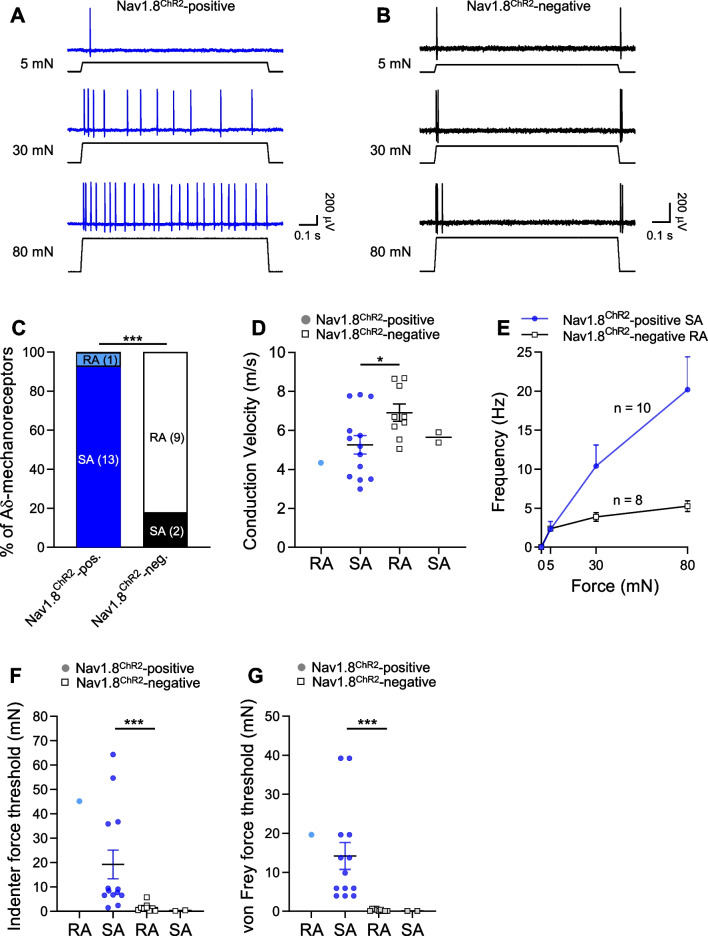
Comparison of properties between Nav1.8^ChR2^-positive and Nav1.8^ChR2^-negative Aδ-fiber mechanoreceptors. **A**, **B** Two sets of sample traces show AP impulses evoked by indentations (5, 30, and 80 mN) in a Nav1.8^ChR2^-positive Aδ-fiber SA-mechanoreceptor (**A**) and a Nav1.8^ChR2^-negative Aδ-fiber RA-mechanoreceptors (**B**). **C** Percent of Nav1.8^ChR2^-positive and Nav1.8^ChR2^-negative Aδ-fiber mechanoreceptors that display SA impulses or RA impulses. Recordings numbers are indicated in the bars. **D** Conduction velocity of Nav1.8^ChR2^-positive and Nav1.8^ChR2^-negative Aδ-fiber mechanoreceptors. **E** Frequency of AP impulses evoked by different forces (5, 30, and 80 mN) in Nav1.8^ChR2^-positive Aδ-fiber SA-mechanoreceptors and Nav1.8^ChR2^-negative Aδ-fiber RA-mechanoreceptors. **F**, **G** Indenter (**F**) and von Frey (**G**) force thresholds for evoking AP impulses in Nav1.8^ChR2^-positive Aδ-fiber RA-mechanoreceptors (n = 1), Nav1.8^ChR2^-positive Aδ-fiber SA-mechanoreceptors (n = 13), Nav1.8^ChR2^-negative Aδ-fiber RA-mechanoreceptors (n = 9), and Nav1.8^ChR2^-negative Aδ-fiber SA-mechanoreceptors (n = 2). Data represent individual observations and/or mean ± SEM, *p < 0.05, ***p < 0.001

Properties of Nav1.8^ChR2^-positive and Nav1.8^ChR2^-negative Aδ-fiber mechanoreceptors were compared. Ramp-and-hold mechanical stimulation with indenter evoked AP impulses at the receptive field of both Nav1.8^ChR2^-positive (Fig. 4A, C) and Nav1.8^ChR2^-negative Aδ-fiber mechanoreceptors (Fig. 4B, C). Almost all the Nav1.8^ChR2^-positive Aδ-fiber mechanoreceptors (n = 13/14, 93%) displayed SA impulses in response to mechanical indentation stimulation (Fig. 4A, C). In contrast, most Nav1.8^ChR2^-negative Aδ-fiber mechanoreceptors (n = 9/11, 82%) showed RA responses (Fig. 4B, C), and only a small fraction (n = 2/11, 18%) of Nav1.8^ChR2^-negative Aδ-fiber mechanoreceptors displayed SA impulses in response to the sustained mechanical stimulation. The patterns of AP impulses were significantly different between Nav1.8^ChR2^-positive and Nav1.8^ChR2^-negative Aδ-fiber mechanoreceptors (p < 0.001, Fig. 4C). We compared conduction velocity between Nav1.8^ChR2^-positive Aδ-fiber SA-mechanoreceptors and Nav1.8^ChR2^-negative Aδ-fiber RA-mechanoreceptors (Fig. 4D). The conduction velocity of Nav1.8^ChR2^-positive Aδ-fiber SA-mechanoreceptors was 5.3 ± 0.5 m/s (n = 13), significantly slower than the Nav1.8^ChR2^-negative Aδ-fiber RA-mechanoreceptors (6.9 ± 0.4 m/s, n = 9, p < 0.05) (Fig. 4D). Although presented (Fig. 4D), Nav1.8^ChR2^-positive Aδ-fiber RA-mechanoreceptors (n = 1) and Nav1.8^ChR2^-negative Aδ-fiber SA-mechanoreceptors (n = 2) had too small sample sizes to allow for any meaningful statistical comparison. Nav1.8^ChR2^-positive Aδ-fiber SA-mechanoreceptors displayed large enhancement of AP impulse frequency with increased mechanical stimulation forces up to 80 mN (Fig. 4E). On the other hand, Nav1.8^ChR2^-negative Aδ-fiber RA-mechanoreceptors showed minimal enhancement of AP impulse frequency with increased mechanical stimulation forces (Fig. 4E).

Nav1.8^ChR2^-positive Aδ-fiber SA-mechanoreceptors showed significantly higher mechanical thresholds in comparison with Nav1.8^ChR2^-negative Aδ-fiber RA-mechanoreceptors as tested by both mechanical indenter (Fig. 4F) and von Frey filaments (Fig. 4G). For the test with mechanical indenter, the force threshold of Nav1.8^ChR2^-positive Aδ-fiber SA-mechanoreceptors was 19.3 ± 5.9 mN (n = 13), significantly higher than Nav1.8^ChR2^-negative Aδ-fiber RA-mechanoreceptors (1.6 ± 0.6 mN, n = 9, p < 0.001) (Fig. 4F). For the test with von Frey filaments, the force threshold of Nav1.8^ChR2^-positive SA Aδ-fiber SA-mechanoreceptors was 14.2 ± 3.4 mN, (n = 13), significantly higher than Nav1.8^ChR2^-negative Aδ-fiber RA-mechanoreceptors (0.3 ± 0.1 mN, n = 9, p < 0.001) (Fig. 4G). Although the mechanical thresholds were presented in Fig. 4F and G, Nav1.8^ChR2^-positive Aδ-fiber RA-mechanoreceptors (n = 1) and Nav1.8^ChR2^-negative Aδ-fiber SA-mechanoreceptors (n = 2) had too small sample sizes to allow for any meaningful statistical comparison.

### Properties of Nav1.8^ChR2^-positive C-fiber mechanoreceptors

**Fig. 5:**
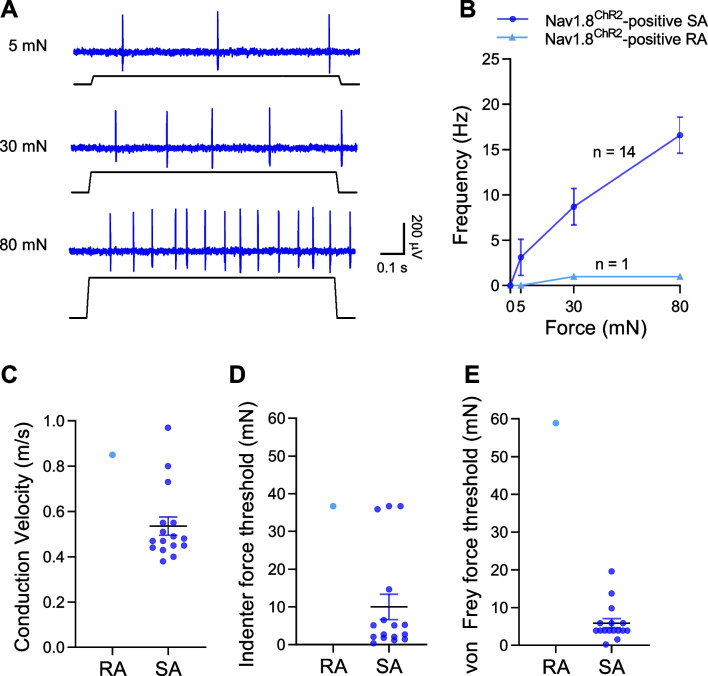
Properties of Nav1.8^ChR2^-positive C-fiber mechanoreceptors. **A** Sample traces show SA impulses evoked by indentations (5, 30, 80 mN) in a Nav1.8^ChR2^-positive C-fiber SA-mechanoreceptor. **B** Frequency of impulses evoked by different indentation forces in Nav1.8^ChR2^-positive C-fiber SA-mechanoreceptors (n = 14) and a Nav1.8^ChR2^-positive C-fiber RA-mechanoreceptor (n = 1). **C** Conduction velocity of Nav1.8^ChR2^-positive C-fiber SA-mechanoreceptors (n = 16) and a Nav1.8^ChR2^-positive C-fiber RA-mechanoreceptor (n = 1). **D**, **E** Indenter (**D**) and von Frey (**E**) force thresholds for evoking AP impulses in Nav1.8^ChR2^-positive C-fiber SA-mechanoreceptors (n = 16) and a Nav1.8^ChR2^-positive C-fiber RA-mechanoreceptor (n = 1). Data represent individual observations and/or mean ± SEM

All recorded C-fiber mechanoreceptors were found to be Nav1.8^ChR2^-positive (Fig. 2F). Of 15 Nav1.8^ChR2^-positive C-fiber mechanoreceptors tested with graded force stimulation, 14 of them displayed SA impulses in response to the ramp-and-hold mechanical stimulation, and AP impulses enhanced with the increased mechanical indentation force (n = 14). Only one Nav1.8^ChR2^-positive C-fiber mechanoreceptors showed RA impulses (Fig. 5B). Conduction velocity of Nav1.8^ChR2^-positive C-fiber SA-mechanoreceptors of the above 14 recordings and two other recordings not included in Fig. 5B was 0.53 ± 0.04 m/s (n = 16), and the single Nav1.8^ChR2^-positive C-fiber RA-mechanoreceptor had the conduction velocity of 0.85 m/s (n = 1) (Fig. 5C). Tested with the mechanical indenter, Nav1.8^ChR2^-positive C-fiber SA-mechanoreceptors had a force threshold of 10.0 ± 3.4 mN (n = 16) (Fig. 5D). The single Nav1.8^ChR2^-positive C-fiber RA-mechanoreceptor had a force threshold of 36.7 mN (Fig. 5D). Tested with von Frey filaments, the Nav1.8^ChR2^-positive C-fiber SA-mechanoreceptors had a force threshold of 5.9 ± 1.2 mN (n = 16, Fig. 5E). The single Nav1.8^ChR2^-positive C-fiber RA-mechanoreceptor had a force threshold of 58.8 mN.

### Comparison of mechanical sensitivity among Nav1.8^ChR2^-positive Aβ-, Aδ-, and C-fiber mechanoreceptors

**Fig. 6:**
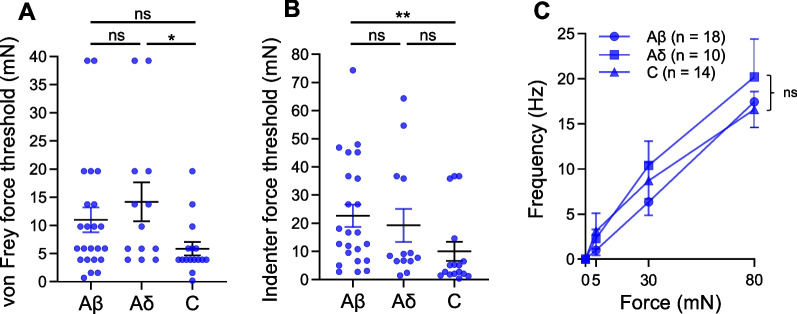
Comparison of mechanosensitivity among Nav1.8^ChR2^-positive Aβ-fiber mechanoreceptors, Aδ-fiber mechanoreceptors, and C-fiber mechanoreceptors. **A** Comparison of von Frey threshold of Nav1.8^ChR2^-positive Aβ-fiber mechanoreceptors, Nav1.8^ChR2^-positive Aδ-fiber mechanoreceptors, and Nav1.8^ChR2^-positive C-fiber mechanoreceptors. **B** Comparison of indenter force threshold of Nav1.8^ChR2^-positive Aβ-fiber mechanoreceptors, Nav1.8^ChR2^-positive Aδ-fiber mechanoreceptors, Nav1.8^ChR2^-positive C-fiber mechanoreceptors. **C** Comparison of impulse frequency evoked by different forces (5, 30 and 80 mN) applied by indenter in Nav1.8^ChR2^-positive Aβ-fiber mechanoreceptors, Nav1.8^ChR2^-positive Aδ-fiber mechanoreceptors, and Nav1.8^ChR2^-positive C-fiber mechanoreceptors. Data are from Figs. 3, 4, 5 and replotted here for comparison. Data represent individual observations and/or mean ± SEM, *p < 0.05, ns, not significantly different

We compared the mechanical thresholds for evoking AP impulses among Nav1.8^ChR2^-positive Aβ-, Aδ-, and C-fiber SA-mechanoreceptors (Fig. 6A). For the experiments using von Frey filaments, the force thresholds were 11.0 ± 2.2 mN (n = 23) for Nav1.8^ChR2^-positive Aβ-fiber SA-mechanoreceptors, 14.2 ± 3.4 mN (n = 13) for Nav1.8^ChR2^-positive Aδ-fiber SA-mechanoreceptors, and 5.9 ± 1.2 mN (n = 16) for Nav1.8^ChR2^-positive C-fiber SA-mechanoreceptors (Fig. 6A). The force thresholds between Nav1.8^ChR2^-positive Aβ-fiber SA-mechanoreceptors and Nav1.8^ChR2^-positive Aδ-fiber SA-mechanoreceptors were not significantly different. The force thresholds between Nav1.8^ChR2^-positive Aβ-fiber SA -mechanoreceptors and Nav1.8^ChR2^-positive C-fiber SA-mechanoreceptors were also not significantly different. The force thresholds were higher in Nav1.8^ChR2^-positive Aδ-fiber SA-mechanoreceptors than in Nav1.8^ChR2^-positive C-fiber SA-mechanoreceptors (p < 0.05, Fig. 6A). With mechanical indenter, the force thresholds were 22.7 ± 4.0 mN (n = 23) for Nav1.8^ChR2^-positive Aβ-fiber SA-mechanoreceptors, 19.3 ± 5.9 mN (n = 13) for Nav1.8^ChR2^-positive SA Aδ-fiber SA-mechanoreceptors, and 10.0 ± 3.4 mN (n = 16) for Nav1.8^ChR2^-positive C-fiber SA-mechanoreceptors (Fig. 6B). The indenter force thresholds between Nav1.8^ChR2^-positive Aβ-fiber SA-mechanoreceptors and Nav1.8^ChR2^-positive Aδ-fiber SA-mechanoreceptors were not significantly different. The indenter force thresholds between Nav1.8^ChR2^-positive Aδ-fiber SA-mechanoreceptors and Nav1.8^ChR2^-positive C-fiber SA-mechanoreceptors were also not significantly different. The force thresholds were higher in Nav1.8^ChR2^-positive Aβ-fiber SA-mechanoreceptors than in Nav1.8^ChR2^-positive C-fiber SA-mechanoreceptors (p < 0.01, Fig. 6B). It was noted that von Frey force threshold for most Nav1.8^ChR2^-positive mechanoreceptors showed threshold over 4 mN, which could be considered as HTMRs. On the other hand, a small portion of Nav1.8^ChR2^-positive mechanoreceptors displayed force threshold below 4 mN (Fig. 6A), which could be considered as LTMRs. With the above classification, von Frey force threshold showed no significant difference among the three types of Nav1.8^ChR2^-positive HTMRs. We further compared AP impulse frequency in response to increased indentation forces among the Nav1.8^ChR2^-positive Aβ-fiber SA-mechanoreceptors (n = 18), Nav1.8^ChR2^-positive Aδ-fiber SA-mechanoreceptors (n = 10), and Nav1.8^ChR2^-positive C-fiber SA-mechanoreceptors (n = 14) (Fig. 6C). While all three types of Nav1.8^ChR2^-positive mechanoreceptors showed nearly linear enhancement of AP impulse frequency in response to increased indentation forces, there was no significant difference in the frequency-force relationship among the three types of Nav1.8^ChR2^-positive mechanoreceptors (Fig. 6C).

## Discussion

In the present study, we have characterized properties of Nav1.8^ChR2^-positive afferent fiber mechanoreceptors and compared with those of Nav1.8^ChR2^-negative afferent fiber mechanoreceptors in the hindpaw glabrous skin of Nav1.8^ChR2^ mice. Our main findings are that Nav1.8^ChR2^-positive Aβ-, Aδ-, and C-fiber mechanoreceptors are mostly HTMRs. In contrast, Nav1.8^ChR2^-negative Aβ- and Aδ-fiber mechanoreceptors are mostly LTMRs, and no Nav1.8^ChR2^-negative C-fiber mechanoreceptors are encountered in the present study. For Nav1.8^ChR2^-positive Aβ-, Aδ-, and C-fiber mechanoreceptors, almost all of them display SA impulses in response to sustained mechanical stimulation. In comparison, in response to sustained mechanical stimulation, Nav1.8^ChR2^-negative Aβ-fiber mechanoreceptors display both SA and RA impulses and Nav1.8^ChR2^-negative Aδ-fiber mechanoreceptors predominantly show RA impulses. Among Nav1.8^ChR2^-positive Aβ-, Aδ-, and C-fiber mechanoreceptors, thresholds of C-fiber mechanoreceptors are significantly lower than those of A-fibers. Nav1.8^ChR2^-positive Aβ-, Aδ-, and C-fiber SA-mechanoreceptors all encode mechanical stimulation intensities by increases of impulse frequency in a similarly manner. Our results provide new insights into the encoding of low (innocuous) and high (noxious) threshold mechanical stimuli by the optogenetically defined subpopulations of mechanoreceptors in the hindpaw glabrous skin of Nav1.8^ChR2^ mice.

Our behavioral assessment shows that Nav1.8^ChR2^ mice have normal paw withdrawal responses to mechanical stimulation examined by the cotton swab test and the von Frey test. The response frequency measured by the cotton swab test and the von Frey test as well as the 50% von Frey threshold measured by the up-down method are consistent with those of the WT mice shown in previous studies [21]. The 50% von Frey thresholds are above 4 mN, a force above which is consider to be high threshold mechanical forces that may activate mechanonociceptors [12]. We show that light stimulation evokes nocifensive responses in a stimulation intensity-dependent manner. The response frequency is enhanced with increased light stimulation intensity and quickly reaches 100% at the light intensity of 10 mW/mm^2^. However, the nocifensive response scores display graded increases with the light stimulation intensity up to 100 mW/mm^2^. Thus, response frequency and nocifensive scores are not well correlated, which may be because Nav1.8^ChR2^-positive A-afferents are the first responder mainly account for the response frequency and Nav1.8^ChR2^-positive C-afferents may account for graded nocifensive scores.

In the present study, we have characterized properties of Nav1.8^ChR2^-positive and Nav1.8^ChR2^-negative afferent fiber mechanoreceptors in the glabrous skin of the hindpaws using the skin-nerve preparations. We have used pressure-clamped single-fiber recording technique [22, 23] to record AP impulses evoked by mechanical stimulation to mechanoreceptors in the glabrous skin. Pressure-clamped single-fiber recording technique allows us to record both mechanically and optogenetically evoked impulses, and also allows to determine the type of afferents based on their conduction velocity. Different from the teased fiber recordings used previously by many investigators, our recording method is a true single-fiber recording technique particularly suitable for studying properties of opto-tagged afferent fiber mechanoreceptors.

The present study shows that nearly 30% of Aβ-fiber mechanoreceptors are Nav1.8^ChR2^-positive. Among Nav1.8^ChR2^-positive Aβ-fiber mechanoreceptors, most of them have von Frey threshold near or above 4 mN, suggesting that most of them are Aβ-fiber HTMRs or mechanonociceptors. This finding is consistent with previous studies showing the presence of mechanosensitive Aβ-fiber nociceptive [9]. The scattered distribution of the force thresholds may suggest that Nav1.8 ^ChR2^-positive Aβ-afferent fibers with high mechanical threshold may be further divided into different functional subtypes. Nav1.8^ChR2^-positive Aβ-fiber HTMRs display SA impulses and graded increases of impulse frequency with increased mechanical stimulation force intensity. These features may allow Nav1.8^ChR2^-positive Aβ-fiber HTMRs to encode a broad range of high-intensity, noxious mechanical stimuli. It should be noted that a small portion of Nav1.8^ChR2^-positive Aβ-fiber mechanoreceptors have mechanical threshold below 4 mN, suggesting they are Aβ-fiber LTMRs, not mechanonociceptors. Consistently, a previous study has shown that Nav1.8^cre^ are not restricted to nociceptors and they are also expressed in some A- and C-fiber LTMRs [20]. It would be interesting to investigate in future whether the low threshold Nav1.8^cre^-positive Aβ-afferent fibers may be a special type of LTMRs. We show that Nav1.8^ChR2^-negative Aβ-fiber mechanoreceptors mostly display low mechanical threshold and thereby are LTMRs. A large portion of Nav1.8^ChR2^-negative Aβ-LTMRs displays SA impulses but a small portion of them shows RA impulses. These Nav1.8^ChR2^-negative Aβ-fiber SA-LTMRs and RA-LTMRs are most likely Merkel cell-neurite complex and Meissner’s corpuscles, respectively, in the glabrous skin of the hindpaws.

We show that the majority of Aδ-fiber mechanoreceptors are Nav1.8^ChR2^-positive. Among Nav1.8^ChR2^-positive Aδ-fiber mechanoreceptors, all of them have von Frey threshold near or above 4 mN and thereby can be considered as Aδ-fiber HTMRs. Nav1.8^ChR2^-positive Aδ-fiber HTMRs have properties similar to those of Nav1.8^ChR2^-positive Aβ-fiber HTMRs. Nav1.8^ChR2^-positive Aδ-fiber HTMRs and Aβ-fiber HTMRs may be the same population of NPY2r^ChR2^-positive A-fiber mechanonociceptors [4], and are likely to be from large-sized DRG that express CGRP [19]. In contrast to Nav1.8^ChR2^-positive Aδ-fiber mechanoreceptors, Nav1.8^ChR2^-negative Aδ-fiber mechanoreceptors all show very low von Frey threshold and most of them display RA responses. Thus, the properties of the Nav1.8^ChR2^-negative Aδ-fiber LTMRs are consistent with D-hair mechanoreceptors [12]. D-hairs and D-hair mechanoreceptors have recently been identified in the glabrous skin of the hindpaws of C57BL/6J (A) and CBA/J (B) mice and also some North American and African rodent species [25].

Similar to Nav1.8^ChR2^-positive Aβ- and Aδ-fiber mechanoreceptors, majority of Nav1.8^ChR2^-positive C-fiber mechanoreceptors show high thresholds and can be considered as C-fiber HTMRs. Consistently, C-fiber mechanoreceptors in DRGs have been shown to be Nav1.8^ChR2^-positive in Nav1.8^ChR2^ mice [19]. Almost all Nav1.8^ChR2^-positive C-fiber mechanoreceptors show SA impulses in response to sustained mechanical stimulation. The impulse frequency linearly enhances with increased stimulation force intensity. Thus, Nav1.8^ChR2^-positive C-fiber HTMRs have properties similar to those of Nav1.8^ChR2^-positive Aβ- and Aδ-fiber HTMRs and they all are suitable for encoding a broad range of high intensity nociceptive mechanical stimuli. It should be noted that a number of Nav1.8^ChR2^-positive C-fiber mechanoreceptors display very low mechanical threshold, indicating that they are C-fiber LTMRs. This is consistent with an immunochemical study with Nav1.8.^ChR2^-positive DRG neurons [19].

We show that Nav1.8^ChR2^-positive Aβ-, Aδ-, and C-fiber HTMRs display SA impulses in response to sustained mechanical stimulation. This is consistent with the findings that mechanonociceptors are SA-mechanoreceptors [1]. In contrast, sustained light stimulation evokes RA rather than SA impulses in almost all Nav1.8^ChR2^-positive Aβ-fiber HTMRs and most Aδ-fiber HTMRs, and these HTMRs may be the first responders for light-induced pain. Only Nav1.8^ChR2^-positive C-fiber HTMRs display SA impulses in response to sustained light stimulation. These results may suggest that A-fiber HTMRs and C-fiber HTMRs have different intrinsic electrophysiological properties, with most of the former intrinsically firing single AP and the latter firing multiple APs in response to sustained depolarization. This may also suggest that mechanical stimulation may act on cells such as keratinocytes that surround the afferent terminals of A-fiber HTMRs to tune the afferent terminals to fire multiple APs in response to sustained mechanical stimulation. Consist with this idea, keratinocytes have been indicated to be involved in mechanotransduction [16, 17]. It would be highly interesting in future to uncover the underlying mechanisms by which Nav1.8^ChR2^-positive Aβ-, Aδ-, and C-fiber HTMRs generate SA impulses in response to sustained mechanical stimulation and also to identify molecular sensors of Aβ-, Aδ-, and C-fiber HTMRs.

## Data Availability

All data generated or analyzed during this study are available from corresponding author on reasonable request.

## Abbreviations

AP: Action potential
CGRP: Calcitonin gene-related peptide
ChR2: Channel rhodopsin 2
CV: Conduction velocity
DRG: Dorsal root ganglion
LTMR: Low threshold mechanoreceptor
HTMR: High threshold mechanoreceptor
Mrgprd: MAS related GPR family member D
RA: Rapidly adapting
SA: Slowly adapting
TRPV1: Transient receptor potential cation channel subfamily V member 1

## References

[1] Abraira VE, Ginty DD (2013). The sensory neurons of touch. Neuron.

[2] Akopian AN, Sivilotti L, Wood JN (1996). A tetrodotoxin-resistant voltage-gated sodium channel expressed by sensory neurons. Nature.

[3] Akopian AN, Souslova V, England S, Okuse K, Ogata N, Ure J, Smith A, Kerr BJ, McMahon SB, Boyce S, Hill R, Stanfa LC, Dickenson AH, Wood JN (1999). The tetrodotoxin-resistant sodium channel SNS has a specialized function in pain pathways. Nat Neurosci.

[4] Arcourt A, Gorham L, Dhandapani R, Prato V, Taberner FJ, Wende H, Gangadharan V, Birchmeier C, Heppenstall PA, Lechner SG (2017). Touch receptor-derived sensory information alleviates acute pain signaling and fine-tunes nociceptive reflex coordination. Neuron.

[5] Bai L, Lehnert BP, Liu JW, Neubarth NL, Dickendesher TL, Nwe PH, Cassidy C, Woodbury CJ, Ginty DD (2015). Genetic identification of an expansive mechanoreceptor sensitive to skin stroking. Cell.

[6] Basbaum AI, Bautista DM, Scherrer G, Julius D (2009). Cellular and molecular mechanisms of pain. Cell.

[7] Cavanaugh DJ, Lee HS, Lo LC, Shields SD, Zylka MJ, Basbaum AI, Anderson DJ (2009). Distinct subsets of unmyelinated primary sensory fibers mediate behavioral responses to noxious thermal and mechanical stimuli. Proc Natl Acad Sci USA.

[8] Chaplan SR, Bach FW, Pogrel JW, Chung JM, Yaksh TL (1994). Quantitative assessment of tactile allodynia in the rat paw. J Neurosci Methods.

[9] Djouhri L, Lawson SN (2004). Abeta-fiber nociceptive primary afferent neurons: a review of incidence and properties in relation to other afferent A-fiber neurons in mammals. Brain Res Brain Res Rev.

[10] Garrison SR, Dietrich A, Stucky CL (2012). TRPC1 contributes to light-touch sensation and mechanical responses in low-threshold cutaneous sensory neurons. J Neurophysiol.

[11] Handler A, Ginty DD (2021). The mechanosensory neurons of touch and their mechanisms of activation. Nat Rev Neurosci.

[12] Koltzenburg M, Stucky CL, Lewin GR (1997). Receptive properties of mouse sensory neurons innervating hairy skin. J Neurophysiol.

[13] Le Pichon CE, Chesler AT. The functional and anatomical dissection of somatosensory subpopulations using mouse genetics. Front Neuroanatomy. 2014;8. 10.3389/fnana.2014.00021 PMC4001001 24795573

[14] Li LS, Rutlin M, Abraira VE, Cassidy C, Kus L, Gong SC, Jankowski MP, Luo WQ, Heintz N, Koerber HR, Woodbury CJ, Ginty DD (2011). The functional organization of cutaneous low-threshold mechanosensory neurons. Cell.

[15] Luo WQ, Enomoto H, Rice FL, Milbrandt J, Ginty DD (2009). Molecular identification of rapidly adapting mechanoreceptors and their developmental dependence on ret signaling. Neuron.

[16] Mikesell AR, Isaeva O, Moehring F, Sadler KE, Menzel AD, Stucky CL. Keratinocyte PIEZO1 modulates cutaneous mechanosensation. Elife. 2020;11. 10.7554/eLife.65987 PMC9512397 36053009

[17] Moehring F, Cowie AM, Menzel AD, Weyer AD, Grzybowski M, Arzua T, Geurts AM, Palygin O, Stucky CL. Keratinocytes mediate innocuous and noxious touch via ATP-P2X4 signaling. Elife. 2018; 7. 10.7554/eLife.31684 PMC5777822 29336303

[18] Neubarth NL, Emanuel AJ, Liu Y, Springel MW, Handler A, Zhang Q, Lehnert BP, Guo C, Orefice LL, Abdelaziz A, DeLisle MM, Iskols M, Rhyins J, Kim SJ, Cattel SJ, Regehr W, Harvey CD, Drugowitsch J, Ginty DD (2020). Meissner corpuscles and their spatially intermingled afferents underlie gentle touch perception. Science.

[19] Patil MJ, Hovhannisyan AH, Akopian AN (2018). Characteristics of sensory neuronal groups in CGRP-cre-ER reporter mice: comparison to Nav1.8-cre, TRPV1-cre and TRPV1-GFP mouse lines. PLoS ONE.

[20] Shields SD, Ahn HS, Yang Y, Han CY, Seal RP, Wood JN, Waxman SG, Dib-Hajj SD (2012). Na(v)1.8 expression is not restricted to nociceptors in mouse peripheral nervous system. Pain.

[21] Smith AK, O’Hara CL, Stucky CL. Mechanical sensitization of cutaneous sensory fibers in the spared nerve injury mouse model. Mol Pain. 2013;9. 10.1186/1744-8069-9-61 PMC3906996 24286165

[22] Sonekatsu M, Gu JG (2019). Functional properties of mechanoreceptors in mouse whisker hair follicles determined by the pressure-clamped single-fiber recording technique. Neurosci Lett.

[23] Sonekatsu M, Yamada H, Gu JG (2020). Pressure-clamped single-fiber recording technique: a new recording method for studying sensory receptors. Mol Pain.

[24] Stirling LC, Forlani G, Baker MD, Wood JN, Matthews EA, Dickenson AH, Nassar MA (2005). Nociceptor-specific gene deletion using heterozygous Na(v)1.8-Cre recombinase mice. Pain.

[25] Walcher J, Ojeda-Alonso J, Haseleu J, Oosthuizen MK, Rowe AH, Bennett NC, Lewin GR (2018). Specialized mechanoreceptor systems in rodent glabrous skin. J Physiol-London.

[26] Wellnitz SA, Lesniak DR, Gerling GJ, Lumpkin EA (2010). The regularity of sustained firing reveals two populations of slowly adapting touch receptors in mouse hairy skin. J Neurophysiol.

[27] Zimmerman A, Bai L, Ginty DD (2014). The gentle touch receptors of mammalian skin. Science.

